# ExsE Is a Negative Regulator for T3SS Gene Expression in *Vibrio alginolyticus*

**DOI:** 10.3389/fcimb.2016.00177

**Published:** 2016-12-06

**Authors:** Jinxin Liu, Shao-Yeh Lu, Lisa H. Orfe, Chun-Hua Ren, Chao-Qun Hu, Douglas R. Call, Johannetsy J. Avillan, Zhe Zhao

**Affiliations:** ^1^Institute of Marine Biology, College of Oceanography, Hohai UniversityNanjing, China; ^2^Paul G. Allen School for Global Animal Health, Washington State UniversityPullman, WA, USA; ^3^Key Laboratory of Tropical Marine Bio-resources and Ecology, Guangdong Provincial Key Laboratory of Applied Marine Biology, South China Sea Institute of Oceanology, Chinese Academy of SciencesGuangzhou, China

**Keywords:** ExsE, negative regulator, T3SS, *Vibrio alginolyticus*, gene expression, ExsACDE

## Abstract

Type III secretion systems (T3SSs) contribute to microbial pathogenesis of *Vibrio* species, but the regulatory mechanisms are complex. We determined if the classic ExsACDE protein-protein regulatory model from *Pseudomonas aeruginosa* applies to *Vibrio alginolyticus*. Deletion mutants in *V. alginolyticus* demonstrated that, as expected, the T3SS is positively regulated by ExsA and ExsC and negatively regulated by ExsD and ExsE. Interestingly, deletion of *exsE* enhanced the ability of *V. alginolyticus* to induce host-cell death while cytotoxicity was inhibited by *in trans* complementation of this gene in a wild-type strain, a result that differs from a similar experiment with *Vibrio parahaemolyticus* ExsE. We further showed that ExsE is a secreted protein that does not contribute to adhesion to Fathead minnow epithelial cells. An *in vitro* co-immunoprecipitation assay confirmed that ExsE binds to ExsC to exert negative regulatory effect on T3SS genes. T3SS in *V. alginolyticus* can be activated in the absence of physical contact with host cells and a separate regulatory pathway appears to contribute to the regulation of ExsA. Consequently, like ExsE from *P. aeruginosa*, ExsE is a negative regulator for T3SS gene expression in *V. alginolyticus*. Unlike the *V. parahaemolyticus* orthologue, however, deletion of *exsE* from *V. alginolyticus* enhanced *in vitro* cytotoxicity.

## Introduction

The type III secretion system (T3SS) is an important virulence-associated surface structure of many Gram-negative pathogens, where it functions to translocate bacterial effector proteins across the bacterial and host membranes directly into the cytosol of host cells (Tseng et al., 2009). T3SSs are composed of a secretion apparatus, translocation apparatus and effector proteins (Hueck, 1998). Normally the genes encoding these proteins are not expressed unless cultured in defined media or in contact with host cells (Hueck, 1998). The regulatory pathway can be remarkably complex and usually involves a series of interacting proteins (Yahr and Wolfgang, 2006; Hauser, 2009).

Transcription of T3SS genes in *Pseudomonas aeruginosa* is controlled by ExsA, which is a member of the AraC/XyIS family of transcriptional regulators (Yahr and Wolfgang, 2006; Hauser, 2009). The transcriptional activity of ExsA is regulated by three additional interacting proteins: ExsC, ExsD, and ExsE (Yahr and Wolfgang, 2006). ExsD is an “anti-activator” that binds ExsA to prevent ExsA-dependent binding to T3SS promoter sequences (Mccaw et al., 2002). ExsC functions as an “anti-anti-activator” by binding directly to ExsD thereby preventing ExsD-ExsA interactions (Dasgupta et al., 2004). ExsC interacts with ExsE, a protein to which ExsC binds with greater affinity than ExsD (Rietsch et al., 2005). Under inducing conditions, ExsE is exported through T3SSs into either medium or host cells thereby allowing a cascade of interactions that frees ExsA to initiate T3SS transcription (Rietsch et al., 2005; Urbanowski et al., 2007).

Two different *Vibrio* T3SSs were originally described (T3SS1 and T3SS2) from a clinical strain of *V. parahaemolyticus* and the T3SS1 shares many characteristics with that of *Yersinia* and *Pseudomonas* (Makino et al., 2003; Troisfontaines and Cornelis, 2005). Subsequently, the mechanism of transcriptional control of T3SS1 genes in *V. parahaemolyticus* was shown to be similar to the *P. aeruginosa* T3SS regulatory pathway (Zhou et al., 2008, 2010; Kodama et al., 2010; Erwin et al., 2012). For example, T3SS1 genes in *V. parahaemolyticus* are positively regulated by ExsA and negatively regulated by ExsD (Zhou et al., 2008) while ExsC can directly bind ExsD to free ExsA and permit the expression of T3SS1 genes (Zhou et al., 2010). VP1702 is a functional equivalent of *P. aeruginosa* ExsE and this orthologue exerts a negative regulatory effect on the production of T3SS1-related proteins (Kodama et al., 2010). Interestingly, a later study revealed that deletion of *exsE* in a different *V. parahaemolyticus* strain, NY-4, showed no apparent impact on the synthesis of T3SS1 proteins and the Δ*exsE* strain was not cytotoxic based on a host-cell infection model (Erwin et al., 2012). The differences in transcriptional regulation of T3SS1 for different *V. parahaemolyticus* strains suggests that regulation of T3SSs may have diverged between genetic lineages of *V*. *parahaemolyticus* and may be divergent between *Vibrio* species as well.

*Vibrio alginolyticus* is widely distributed as the part of the normal microbial flora in marine environments (Zhao et al., 2010). It is also an opportunistic pathogen to people and causes otitis, conjunctivitis, superficial pyodermatitis, gastroenteritis, and life-threating infections in immunocompromised patients (Chien et al., 2002; Campanelli et al., 2008). *V. alginolyticus* is prevalent in coastal waters of southern China and it is commonly associated with costly disease of aquatic animals (Austin, 2010). Previous studies demonstrated that *V. alginolyticus* induces apoptosis, cell rounding and osmotic lysis in fish cells and autophagy in mammalian cell lines in a T3SS-dependent manner (Zhao et al., 2010, 2011). Although the T3SS of *V. alginolyticus* is similar to T3SS1 of *V. parahaemolyticus* with respect to gene synteny (Zhao et al., 2010), it is unclear if the same regulatory mechanism is employed by *V. alginolyticus*.

In this study, we found that expression of T3SS genes in *V. alginolyticus*, like that in *V. parahaemolyticus*, is positively regulated by ExsA and ExsC, and negatively regulated by ExsD. ExsE also functions as a negative regulator, which is consistent with previous studies (Rietsch et al., 2005; Kodama et al., 2010; Erwin et al., 2012). One important difference, however, is that the deletion of *exsE* enhanced the *in vitro* cytotoxicity of *V. alginolyticus*, an outcome that is distinct from the outcome of deleting *exsE* from *V. parahaemolyticus* strain NY-4 (Erwin et al., 2012).

## Materials and methods

### Bacterial strains, plasmids, and growth conditions

*Vibrio alginolyticus* strains, including wild-type strain ZJO and associated deletion mutants (Table 1), were routinely grown in trypticase soy broth (TSB) or on 1.5% TSB agar plates (TSA) at 30°C. *E. coli* S17λ*pir* was used in gene deletion experiments and was cultured in LB medium. Expression vector pMMB207 (Morales et al., 1991) was used in complementation experiments and suicide plasmid pDM4 (Milton et al., 1996) was used to generate gene knockouts. Vector pACYCDuet-1 (Novagen) was used to examine protein-protein interactions. When appropriate, ampicillin (100 μg mL^−1^) or chloramphenicol (34 μg mL^−1^) was added.

**Table 1:** **Strains and plasmids used in this study**.

**Strains and plasmids**	**Relevant characteristics**	**Sources**
***E. COLI***		
S17-1λ*pir*	*thi pro hsdR hsdM^+^ recA* RP4-2-Tc::Mu-Km::Tn7λ*pir*	Milton et al., 1992
BL21 (DE3)	F^−^*ompT hsdS_B_* (${\text{r}}_{\text{B}}^{-}{\text{m}}_{\text{B}}^{-}$) *gal dcm* (DE3)	Novagen
S17_pDM4_*exsA*_A1F+A2R	S17 carrying pDM4_*exsA*_A1F+A2R	This study
S17_pDM4_*exsC*_A1F+A2R	S17 carrying pDM4_*exsC*_A1F+A2R	This study
S17_pDM4_*exsD*_A1F+A2R	S17 carrying pDM4_*exsD*_A1F+A2R	This study
S17_pDM4_*exsE*_A1F+A2R	S17 carrying pDM4_*exsE*_A1F+A2R	This study
S17_pMMB207_*exsA*_His	S17 carrying pMMB207_*exsA*_His	This study
S17_pMMB207_*exsC*_His	S17 carrying pMMB207_*exsC*_His	This study
S17_pMMB207_*exsD*_His	S17 carrying pMMB207_*exsD*_His	This study
S17_pMMB207_*exsE*_His	S17 carrying pMMB207_*exsE*_His	This study
S17_pDM4_*exsA*_HA_Insertion	S17 carrying pDM4_*exsA*_HA_Insertion	This study
BL21_pACYCDuet-1_*exsA*	BL21 carrying pACYCDuet-1_*exsA*	This study
BL21_pACYCDuet-1_*exsC*	BL21 carrying pACYCDuet-1_*exsA*	This study
BL21_pACYCDuet-1_*exsD*	BL21 carrying pACYCDuet-1_*exsD*	This study
BL21_pACYCDuet-1_*exsE*	BL21 carrying pACYCDuet-1_*exsE*	This study
***V. ALGINOLYTICUS***		
ZJO	Opaque variant of wild strain ZJ51; isolated from diseased grouper fish off the Southern China coast; Ap^r^	Chen et al., 2009
Δ*vscC:pexsE*	*In trans* complementation of *exsE* in a T3SS dysfunctional strain (Δ*vscC*); Ap^r^	Zhao et al., 2010
Δ*exsA*	*exsA* deletion mutant; Ap^r^	This study
Δ*exsA*: p*exsA*	Δ*exsA* complemented *in trans* with wild-type *exsA* gene located in the plasmid of pMMB207; Ap^r^	This study
ZJO: p*exsA*	Wild-type strain complemented *in trans* with wild-type *exsA* gene located in the plasmid of pMMB207; Ap^r^	This study
Δ*exsC*	*exsC* deletion mutant; Ap^r^	This study
Δ*exsC*: p*exsC*	Δ*exsC* complemented *in trans* with wild-type *exsC* gene located in the plasmid of pMMB207; Ap^r^	This study
ZJO: p*exsC*	Wild-type strain complemented *in trans* with wild-type *exsC* gene located in the plasmid of pMMB207; Ap^r^	This study
Δ*exsD*	*exsD* deletion mutant; Ap^r^	This study
Δ*exsD*: p*exsD*	Δ*exsD* complemented *in trans* with wild-type *exsD* gene located in the plasmid of pMMB207; Ap^r^	This study
ZJO: p*exsD*	Wild-type strain complemented *in trans* with wild-type *exsD* gene located in the plasmid of pMMB207; Ap^r^	This study
Δ*exsE*	*exsE* deletion mutant; Ap^r^	This study
Δ*exsE*: p*exsE*	Δ*exsE* complemented *in trans* with wild-type *exsE* gene located in the plasmid of pMMB207; Ap^r^	This study
ZJO: p*exsE*	Wild-type strain complemented *in trans* with wild-type *exsE* gene located in the plasmid of pMMB207; Ap^r^	This study
ZJO_ pDM4_*exsA*_HA_Insertion	Wild-type strain was incorporated a *exsA* gene with a HA tag at the 3′ terminus in the chromosome; Ap^r^Cm^r^	This study
Δ*exsC*_ pDM4_*exsA*_HA_Insertion	Δ*exsC* mutant was incorporated a *exsA* gene with a HA tag at the 3′ terminus in the chromosome; Ap^r^Cm^r^	This study
***V. PARAHAEMOLYTICUS***		
NY4	Clinical isolate O3: K6	Zhou et al., 2008
**PLASMIDS**		
pMMB207	RSF1010 derivative, *IncQ lacI ^q^* Cm^r^ P*tac oriT*	Morales et al., 1991
pDM4	A suicide vector with ori R6K *sacB*; Cm^r^	Milton et al., 1996
pACYCDuet-1	Prokaryotic expression vector with two MCS, each of which is preceded by a T7 promoter/lac operator; Cm^r^	Novagen
pDM4_*exsA*_A1F +A2R	Flanking region sequences of *exsA* cloned into pDM4	This study
pDM4_*exsC*_A1F +A2R	Flanking region sequences of *exsC* cloned into pDM4	This study
pDM4_*exsD*_A1F +A2R	Flanking region sequences of *exsD* cloned into pDM4	This study
pDM4_*exsE*_A1F +A2R	Flanking region sequences of *exsE* cloned into pDM4	This study
pDM4_*exsA*_HA_Insertion	*exsA* coding sequence and sequences for HA tag at the C-terminus cloned into pDM4	This study
pMMB207_*exsA*_His	*exsA* coding sequence and sequences for 6 His amino acids at the C-terminus cloned into pMMB207	This study
pMMB207_*exsC*_His	*exsC* coding sequence and sequences for 6 His amino acids at the C-terminus cloned into pMMB207	This study
pMMB207_*exsD*_His	*exsD* coding sequence and sequences for 6 His amino acids at the C-terminus cloned into pMMB207	This study
pMMB207_*exsE*_His	*exsE* coding sequence and sequences for 6 His amino acids at the C-terminus cloned into pMMB207	This study
pACYCDuet-1_*exsA*	*exsA* coding sequence cloned into pACYCDuet-1	This study
pACYCDuet-1_*exsC*	*exsC* coding sequence cloned into pACYCDuet-1	This study
pACYCDuet-1_*exsD*	*exsD* coding sequence cloned into pACYCDuet-1	This study
pACYCDuet-1_*exsE*	*exsE* coding sequence cloned into pACYCDuet-1	This study

### Construction of deletion mutants

All deletions were made by allelic exchange following a method described previously (Milton et al., 1996). Briefly, primers ExsA_A1_F, ExsA_A1_R, ExsA_A2_F, and ExsA_A2_R (Table 2) were used to amplify two fragments flanking the coding sequence of *exsA*. Both amplified fragments incorporated 10-bp overlapping sequences in addition to BglII and SalI restriction site sequences, respectively. These products were used as templates for “splicing by overlap extension” (SOE) PCR to produce a hybrid product using primers ExsA_A1_F and ExsA_A2_R. The full-length fragment was digested with BglII and SalI and then ligated into the suicide vector pDM4 (digested with the same enzymes), generating pDM4_exsA_A1F+A2R. The resultant plasmid was electroporated into *E. coli* S17-1λ*pir* and transferred into *V. alginolyticus* strain ZJO by conjugation. Successful transconjugants were selected on TSA plates with ampicillin and chloramphenicol. Secondary cross-over was detected by subsequent plating on agar containing 10% sucrose. For this latter selection, strains that successfully excised the plasmid sequence through secondary cross-over no longer propagate the plasmid-encoded *sacB* (Gay et al., 1985) and thus can grow in the presence of sucrose. The *exsA* deletion strain was confirmed by PCR with primers ExsA_int_F and ExsA_int_R and designated as Δ*exsA*. Construction of *exsC, exsD* and *exsE* deletion mutants were performed in the same manner using corresponding primers (Table 2).

**Table 2:** **Primers used in this study**.

**Primer name**	**Sequences (5′-3′)**
ExsA_A1_F	TGAAGATCTTATCTCGCTCCTTGAACAC
ExsA_A1_R	TAGCCACTTGTTTCTACCCTTCATTATTTTGA
ExsA_A2_F	AGGGTAGAAACAAGTGGCTATCGCGAAATGAA
ExsA_A2_R	AGCGTCGACCAGACGAGAGTTGATGTAGT
ExsA_int_F	TGTCGTTCACAATGGTCAG
ExsA_int_R	AGGCACATAATGGCATCAG
ExsC_A1_F	GTCAGAGCTCTCGACCCGTTAGGCTTCT
ExsC_A1_R	CTATGTCAGCGGTGTCTTTATGTCCAATGACA
ExsC_A2_F	TAAAGACACCGCTGACATAGGAATAGTCCC
ExsC_A2_R	GACTCTCGAGGAATAACCCAATAAAACC
ExsC_int_F	ACAATAACGCTTCCCACG
ExsC_int_R	TGTCAGCACGCCAAACTA
ExsD_A1_F	GTCAGAGCTCTGATGCCATTATGTGCCTAA
ExsD_A1_R	GAGGTGATTGTTTATGTTCGTCTCCGCAC
ExsD_A2_F	CGAACATAAACAATCACCTCAGCCAGAT
ExsD_A2_R	GACTCTCGAGCGTTCTTGTTCCAATAATGC
ExsD_int_F	GATAGCAGCACAATCACAAC
ExsD_int_R	GCACTTCCGAACACCAAT
ExsE_A1_F	GTCAGAGCTCTACATTCAGCCAACCATG
ExsE_A1_R	TTTATGTCCAGATATCACAATATAAGCAGG
ExsE_A2_F	TTGTGATATCTGGACATAAAGACACCTAAACTCTC
ExsE_A2_R	GACTCTCGAGCACTGCATCTAACGGAAA
ExsE_int_F	AGTTGCGGATCAAAGTCC
ExsE_int_R	ACACCAATTCATCGGTTC
ExsA-comp-F	AGGATAGAATTCATGGATGTGTCAGGCCAACTA
ExsA-comp-His-R	AGTTAGGGATCCTCAATGGTGATGGTGATGGTGTTTCGCGATAGCCACTTGA
ExsC-comp-F	AGGATAGAATTCATGTCAGCACGCCAAACTATC
ExsC-comp-His-R	AGTTAGGGATCCCTAATGGTGATGGTGATGGTGAACTCTCAAGTCTAAAGTTT
ExsD-comp-F	AGGATAGAATTCATGAAAAAGCAGCATTGGC
ExsD-comp-His-R	AGTTAGGGATCCTTAATGGTGATGGTGATGGTGGATCTGGCTGAGGTGATTGC
ExsE-comp-F	AGGATAGAATTCATGTCCAATGACATCCAATCCA
ExsE-comp-His-R	AGTTAGGGATCCTCAATGGTGATGGTGATGGTGGGAACGTTGAATTATCGCC
VscY_F	GGCGTGTTTACAAAGTGG
VscY_R	TGCCGAGTCAGGATGAAG
VseE_F	ATGAAGGCGAGACGAACA
VseE_R	GCACCCTAAATCCAACTGAC
1687_F	ATGATTGTTGCCATCTACT
1687_R	ACTCGGTTTATTACCTGAA
1686_F	GCAAGCGGTGTTTGATAT
1686_R	ATTGGTTACGCCACTTTT
VopB_F	AGAAGCGGGCGTAAATG
VopB_R	CACCACCAAACGTCACAAC
VopD_F	TCGGGTGTATTAGCGGGTGC
VopD_R	CTCGCCATTTCATTCTTGATTTCT
ExsA_F	AGCACTATGGCATTTCTC
ExsA_R	AACGACGACGGTAACTCT
ExsC_F	TAATCCAGTCGCCTAA
ExsC_R	CTCTATCGCTCTTTCTT
ExsD_F:	CGGAGTACACCTCTACAACC
ExsD_R	TCTTGAACCATTGCCATAC
ExsE_F	GCGTCATACTGCTTTCTG
ExsE_R	ACCAATTCATCGGTTCA
DnaK_F	TAAACCCTGACGAAGC
DanK_R	AGTCATCACGCCACCC
pACYC_ExsA_F	AGGATAGAATTCGATGGATGTGTCAGGCCAACTA
pACYC_ExsA_R	AGTTAGAAGCTTTCATTTCGCGATAGCCACTTGA
pACYC_ExsC_F	AGGATAGAATTCGATGTCAGCACGCCAAACTATC
pACYC_ExsC_R	AGTTAGAAGCTTCTAAACTCTCAAGTCTAAAGTTT
pACYC_ExsD_F	AGGATACGATCGATGAAAAAGCAGCATTGGC
pACYC_ExsD_R	AGTTAGCTCGAGTTAGGCGTAGTCAGGCACGTCGTAAGGATA GATCTGGCTGAGGTGATTGC
pACYC_ExsE_F	AGGATACGATCGATGTCCAATGACATCCAATCCA
pACYC_ExsE_R	AGTTAGCTCGAGTCAGGCGTAGTCAGGCACGTCGTAAGGATA GGAACGTTGAATTATCGCC
ExsA-insertion-F	AGGATAGAGCTCATGGATGTGTCAGGCCAACTA
ExsA-insertion-HA-R	AGTTAGTCTAGATCAGGCGTAGTCAGGCACGTCGTAAGGATATTTCGCGATAGCCACTTGA

### Complementation

To complement the *exsA* gene, primers ExsA-comp-F and ExsA-comp-His-R were used to amplify the complete *exsA* sequence with a 6X histidine sequence added at the C-terminus. The resulting amplicon was digested with EcoRI and BamHI and ligated into the expression vector pMMB207, which had been digested with the same enzymes to generate the plasmid pMMB207_exsA_His. This plasmid was then electroporated into *E. coli* S17-1λ*pir*, resulting in the strain S17_pMMB207_exsA_His, and conjugated into the Δ*exsA* mutant strain and wild-type strain ZJO resulting in Δ*exsA*:p*exsA* and ZJO:p*exsA*, respectively. Construction of expression vectors for *exsC, exsD* and *exsE* was performed in the same manner with corresponding primers (Table 2).

### Infection and lactate dehydrogenase (LDH) assay

Fathead minnow (FHM) epithelial cells were cultured in M199 medium (HyClone) supplemented with 10% (v/v) fetal bovine serum (HyClone) at 28°C. For LDH assays growth media (10% FBS) was replaced by fresh M199 supplemented with 1% FBS prior to infection to reduce background LDH activity. For complementation strains of *V. alginolyticus*, overnight culture was diluted 1:100 into fresh TSB with chloramphenicol and incubated at 30°C with shaking until an OD_600_ of ~0.6 was obtained. Isopropyl β-D-1-thiogalactopyranoside (IPTG; 1 mM) was then added to induce protein expression. After 3 h the induced bacteria were pelleted by centrifugation (3220 × *g*; 30 min) and resuspended in an equivalent volume of M199 with 1% FBS. Wild-type and deletion mutants were also treated in the same manner, but without antibiotics. Bacterial suspensions were added to cell monolayers in a 12-well plate at a multiplicity of infection (m.o.i.) of ~100. Plates were centrifuged at ~600 × *g* for 2 min to synchronize contact with host cells. Supernatants were collected at 1.5 h post infection and LDH activity was measured with a CytoTox 96 Non-Radioactive Cytotoxicity Assay (Promega). Maximum LDH release was achieved by lysis of cells using 10X Lysis Solution and spontaneous LDH release was measured from uninfected cells. Percent cytotoxicity was calculated as follows:

$$\begin{array}{l} \%\text{Cytotoxicity}=\frac{\text{Test LDH release }–\text{ Spontaneous release}}{\text{Maximum release }–\text{ Spontaneous release}}\times 100 \end{array}$$

### RNA isolation and quantitative RT-PCR (qPCR)

For bacteria grown in TSB, overnight culture was directly inoculated (1:100) into fresh TSB media with appropriate antibiotics and incubated for 3 h. An overnight bacterial culture was also used to infect FHM cell monolayers with an m.o.i. of 100 for 3 h. A total of 3 ml of each co-culture was collected and total RNA was isolated using a RiboPure-Bacteria kit (Ambion) followed by a secondary treatment of DNase using TURBO DNA-free kit (Ambion). Isolated RNA was reverse-transcribed into cDNA using an iScript Reverse Transcription Supermix (Bio-Rad). qPCR was performed with primer pairs (Table 2) to amplify internal fragments using SsoAdvanced SYBR Green Supermix (Bio-Rad) according to manufacturer's instructions. Cycling parameters were identical for all primer sets: 95°C for 30 s, 39 cycles of 95°C for 5 s, 55°C for 15 s, and 72°C for 30 s. Reactions were performed using the CFX 96 real-time PCR system (Bio-Rad) and relative expression was calculated using theΔΔCt method with *dnaK* serving as a house keeping gene (Livak and Schmittgen, 2001; Erwin et al., 2012; Nydam et al., 2014).

### Adhesion assay

Adhesion assays were performed as described previously (Erwin et al., 2012). Briefly, FHM cell monolayers were grown on glass coverslips (Nunc; washed and sterilized) in six-well plates in the presence of M199 supplemented with 10% (v/v) FBS. Monolayers were then inoculated with indicated strains at an m.o.i. ~100. Importantly, *V. alginolyticus* strain ZJO does not adhere to glass coverslips unless host cells are present. We quantified the adherent bacteria by calculating the ratio of bacteria attached to coverslips to total bacteria as previously reported (Letourneau et al., 2011). Results were presented as average percentages from three independent replicates.

### Swarming assay

We prepared semi-solid swarming motility agar (Niu et al., 2005) using LBS [2.5% (w/v) NaCl in LB] broth with the addition of 0.5% (w/v) agar, 0.04% (v/v) sterile Tween 80 and supplemented with ampicillin. A single isolate was selected for each strain and inoculated onto a freshly prepared swarm agar plate. Plates were incubated at 30°C for 8 h and images were obtained using ChemiDoc™MP System (Bio-Rad).

### Protein interaction and purification

To examine the potential interactions between ExsA-ExsD, ExsA-ExsE, ExsC-ExsD, and ExsC-ExsE, recombinant proteins (ExsA-His, ExsC-His, ExsD-HA, and ExsE-HA) were induced and expressed in BL21 (DE3) with vector pACYCDuet-1. Overnight bacterial culture was diluted (1:100) into fresh LB media and IPTG (1 mM) was added when the OD_600_ reached ~0.6. After a 5 h incubation at 37°C, samples (~5 mL) were collected and centrifuged for 20 min at ~12,000 × *g*. Supernatant was decanted and the cell pellets were re-suspended in BugBuster Master Mix (1 g pellet in 5 mL reagent; Novagen) to prepare crude protein extracts. Whole-cell lysate was collected and loaded onto Ni^2+^ resin columns (Invitrogen) to allow protein binding overnight at 4°C. Columns were then washed six times with washing buffer (30 mM immidazole) and proteins were eluted with 500 μL elution buffer (300 mM immidazole). Presence of proteins was determined by western blot analysis with monoclonal anti-His antibody (1:2000; Invitrogen) and polyclonal anti-HA antibody (1:2000; Invitrogen). To control for the possibility that HA-tagged protein bound non-specifically to the resin column, ExsD-HA, and ExsE-HA proteins were expressed alone and passed through the Ni^2+^ column to serve as controls for further western blot analysis.

### Trichloroacetic acid (TCA) precipitation assay

Fresh FHM cell culture was prepared 1 day prior to bacterial infection in a 75 cm^2^ cell culture flask (Nunc). Overnight bacterial culture (Δ*exsE*:p*exsE* or Δ*vscC*:p*exsE*) was diluted into fresh TSB media and IPTG (1 mM) was used to induce expression of *exsE* for 5 h. Induced bacteria were used to infect monolayers of FHM cells for 4 h and the bacterial cell pellet was analyzed for the presence of ExsE. Cell culture media (M199) was collected and filtered (0.8 μm, Millipore). TCA (100%) was added to the filtrates (1:4) and incubated for 1 h at 4°C. Supernatant was centrifuged (22,000 × *g*, 30 min) and the protein pellet was washed with cold acetone to remove residual TCA. Air-dried pellets were re-suspended with PBS and samples were analyzed by western blot.

### Western blot

An equal volume of 2X Laemmli sample buffer (Bio-Rad) was added into each sample and all samples were boiled at 100°C for 5 min and loaded onto a 12% SDS-PAGE. After electrophoresis proteins were transferred to a PVDF membrane (Bio-Rad) and the membrane was blocked with 5% skimmed milk in PBS containing 0.05% (v/v) Tween 20 (PBS-T). After 1 h blocking, the membrane was probed with monoclonal anti-His or anti-HA antibody (1:2000; Invitrogen) for 1 h at room temperature. Secondary antibody (anti-mouse-DyLight 488; Thermo Scientific) was diluted 1:5000 in PBS-T with 1% milk for 1 h. Blots were washed 3X with PBS-T and images were obtained using a ChemiDoc™MP System (Bio-Rad).

### Generation of HA-tagged ExsA in the chromosome

An HA-sequence was inserted at the C-terminus of the chromosomally encoded *exsA* as described previously (Zhou et al., 2010). Briefly, the coding sequence of *exsA* with a HA tag was amplified from wild-type *V. alginolyticus* by using primers ExsA-insertion-F and ExsA-insertion-HA-R (Table 2). The resulting amplicon was digested with XbaI and SacI and ligated into suicide vector pDM4 (digested with the same enzymes), resulting in plasmid pDM4_exsA_HA_Insertion. The plasmid was electroporated into *E. coli* S17-1λ*pir*, generating the strain S17_pDM4_exsA_HA_Insertion. Plasmid pDM4_exsA_HA_Insertion was then conjugated into strain ZJO and Δ*exsC* resulting in strains ZJO_ pDM4_exsA_HA_Insertion and ΔexsC_pDM4_exsA_HA_Insertion, respectively. Synthesis of ExsA was determined by western blot analysis using anti-HA antibody as described above.

### Statistical analysis

A paired *t*-test was used for paired comparisons across transcriptional profiles because we were interested in the overall pattern of difference rather than specific differences of individual genes (for deletion mutants, transcriptional results for the knockout gene were excluded for these comparisons). For phenotype comparisons, a Kruskal-Wallis one-way ANOVA was used to assess treatment effects in conjunction with a Tukey-Kramer test for multiple-comparison (α = 0.05). qPCR data were log-transformed for statistical analysis. Because log_10_(0) is undefined, 0.05 was added to zero values before transformation. Western blots were quantified using ImageJ (https://imagej.nih.gov/ij/index.html) for statistical comparison and Dnak was used to normalize band intensity data. Histograms were prepared by using SigmaPlot (ver. 12.5, Systat Software, Inc., San Jose, CA) and heat maps for qPCR data were prepared by using the gplots package in R (version 3.3.1).

## Results

### ExsE exhibits a negative regulatory effect on *V. alginolyticus* T3SS-induced cell death

**Figure 1:**
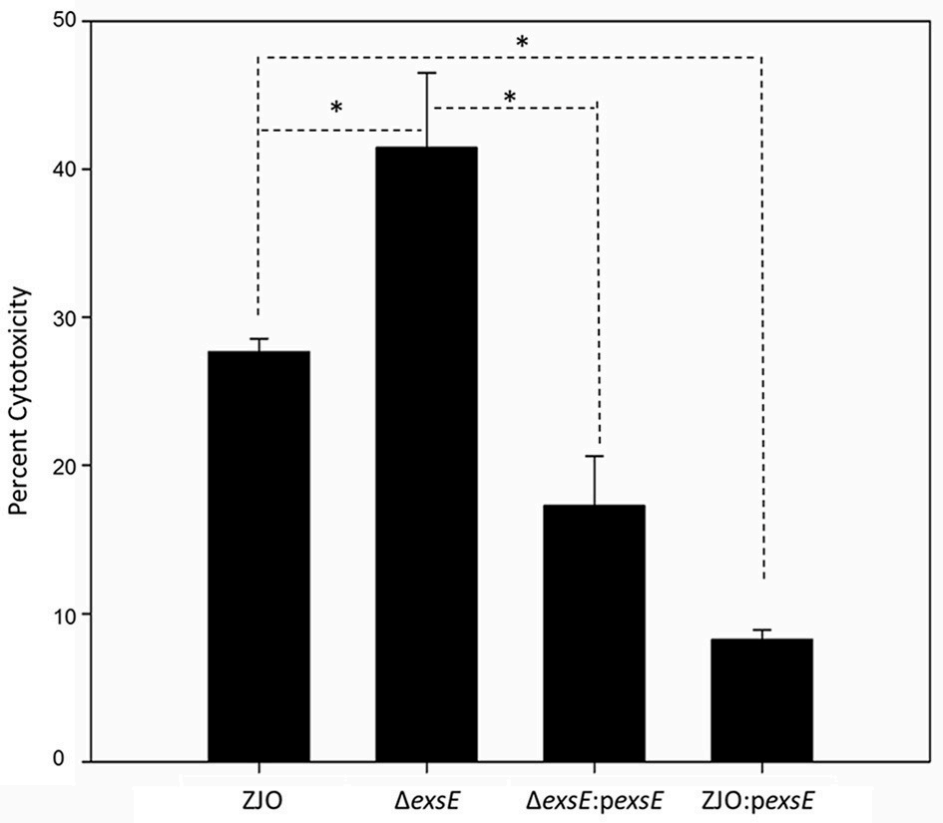
**ExsE exerts a negative regulatory effect on ***in vitro*** cytotoxicity**. LDH assay 1.5 h post infection. Deletion of *exsE* in *V. alginolyticus* led to increased T3SS-induced cell death while intrans expression of *exsE* inhibited cytotoxicity in the wild-type strain. Asterisk represents statistical significance (*P* < 0.05). Error bars represent SEM (*n* = three independent replicates).

To assess the applicability of the ExsACDE regulatory model in T3SS of *V. alginolyticus*, deletion mutants (Δ*exsA*, Δ*exsC*, Δ*exsD*, and Δ*exsE*) were co-cultured with FHM cells. Results from cytotoxicity assays were consistent with ExsA and ExsC functioning as positive regulators, while ExsD functions as a negative regulator for *V. alginolyticus* T3SS (Figure S1). LDH assays revealed that deleting *exsE* from *V. alginolyticus* enhanced cytotoxicity compared with the wild-type strain (Figure 1; *P* < 0.05), consistent with ExsE exhibiting a negative regulatory effect on *V. alginolyticus* T3SS-induced cell death. This conclusion is further supported when overexpression of *exsE* in a wild-type strain resulted in significantly reduced cytotoxicity (Figure 1; *P* < 0.05).

### ExsE is a negative regulator for T3SS gene transcription in *V. alginolyticus*

**Figure 2:**
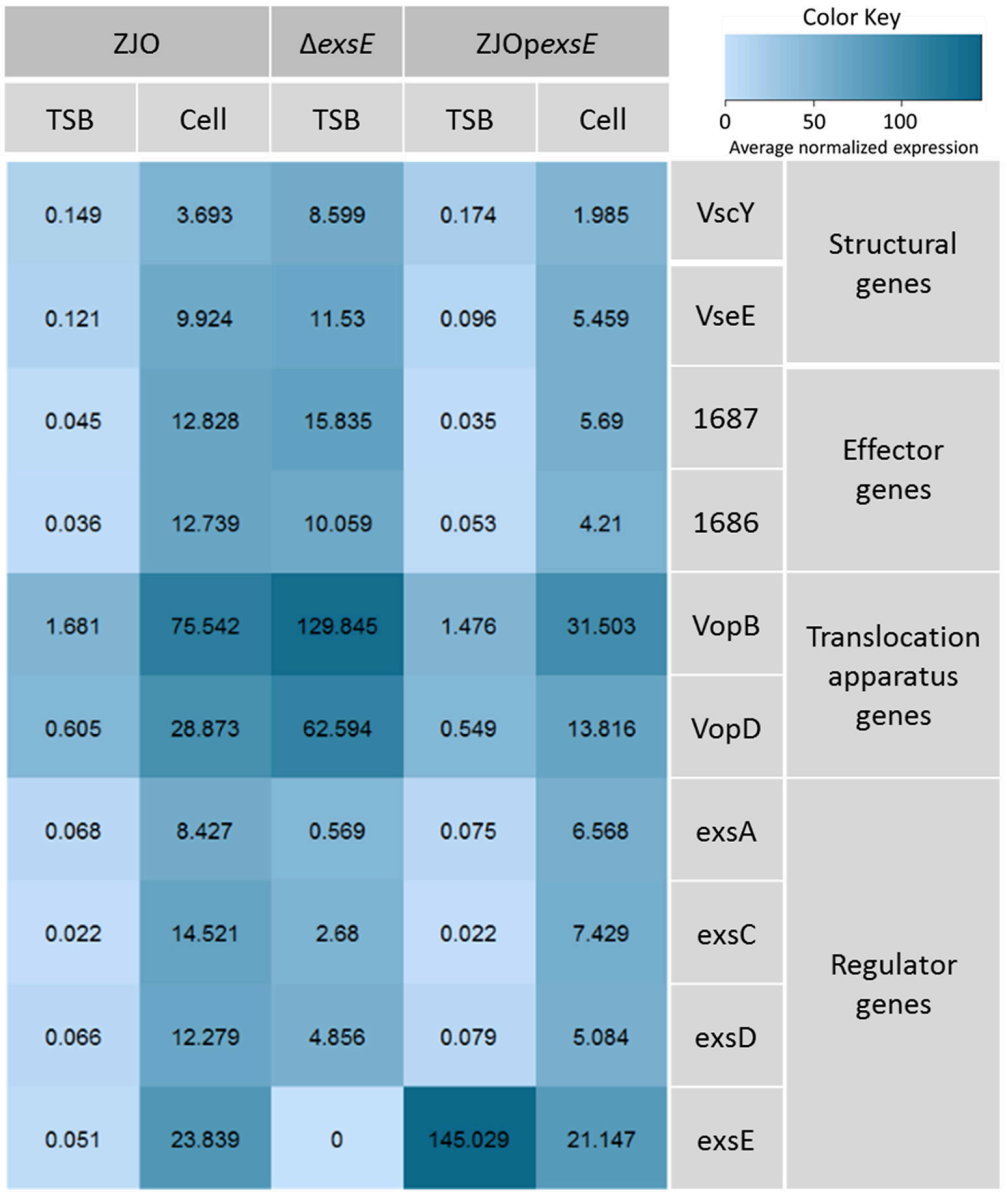
**ExsE is a negative regulator for T3SS genes transcription in ***V. alginolyticus*****. 10 T3SS genes, including structural genes (*vscY* and *vseE*), effector genes (1687 and 1686), translocation genes (*vopB* and *vopD*) and regulatory genes (*exsA, exsC, exsD* and *exsE*), were examined using qPCR to show their transcription pattern in different *V. alginolyticus* strains (ZJO, Δ*exsE* and Δ*exsE*:p*exsE*) in TSB or contact with FHM cells. T3SS genes were transcribed after contact with host cells while transcription is limited under TSB only conditions. Deletion of *exsE* resulted in significantly more T3SS transcription under non-inducing conditions and transcription was inhibited by overexpression of *exsE* in wild-type strain even under inducing conditions. Average normalized expression data were added into individual box in the heatmap.

The type III secretion system (T3SS) genes in strain ZJO are transcribed when cultured with FHM cells, but are not transcribed when cultured in TSB (Figure 2, 20-fold mean increase, *t* = −3.12, 9 df, *P* = 0.012). Hereafter, we refer to FHM cells and TSB as “inducing” and “non-inducing” conditions, respectively. Deletion of *exsE* permitted transcription of T3SS genes under non-inducing conditions (Δ*exsE*, TSB) at levels comparable to the wild-type *V. alginolyticus* (ZJO, cell) strain when cultured under inducing conditions (Figure 2; *t* = −1.02, 8 df, *P* = 0.34). Presumably this occurs because without ExsE, ExsC is free to bind ExsD allowing any ExsA present in the cytoplasm to serve as a transcription regulator even in the absence of cell contact. Notably, under these conditions *exsA* expression was still nearly 15-fold less than what we observed when the wild-type strain was cultured under inducing conditions, which may indicate that protein turnover is relatively slow for ExsA. *In trans* expression of *exsE* (ZJOp*exsE*, cell) reduced overall gene expression by 10.8-fold (*t* = 2.47, 8 df, *P* = 0.039) compared to the wild-type strain under inducing conditions. This is to be expected if ExsE binds ExsC and allows ExsD to interfere with ExsA transcriptional regulator activity. Nevertheless, negative activity was clearly not complete because gene expression for the ZJOp*exsE* strain was still 8.8-fold greater than the wild-type strain under non-inducing conditions (ZJO, TSB; *t* = −3.1, 8 df, *P* = 0.015). Importantly, we also confirmed that knocking out *exsA* completely eliminates T3SS expression as predicted if it is the sole transcriptional regulator (Figure S2, *t* = −0.97, df = 8, *P* = 0.36). Knocking out *exsC* only partially reduced overall expression (Figure S2, mean 6-fold reduction, *t* = −5.46, 8 df, *P* < 0.001) while *in trans* expression of *exsD* in the presence of cells reduced expression relative to the wild-type strain with cells (Figure S2, 11.4-fold reduction, *t* = −2.46, 8 df, *P* = 0.04), but still exhibited significant overall expression relative to the wild-type strain when cultured in TSB alone (9.4-fold increase, *t* = 3.72, 8 df, *P* = 0.006). Collectively, these expression results are consistent with the ExsACDE model of transcriptional control, although positive regulation of *exsA*, and hence up-regulation of the entire T3SS, is probably controlled by a separate transcriptional regulation system as has been hypothesized for *V. parahaemolyticus* (Zhou et al., 2010).

### Evidence for Non-ExsACDE regulation of *exsA*

**Figure 3:**
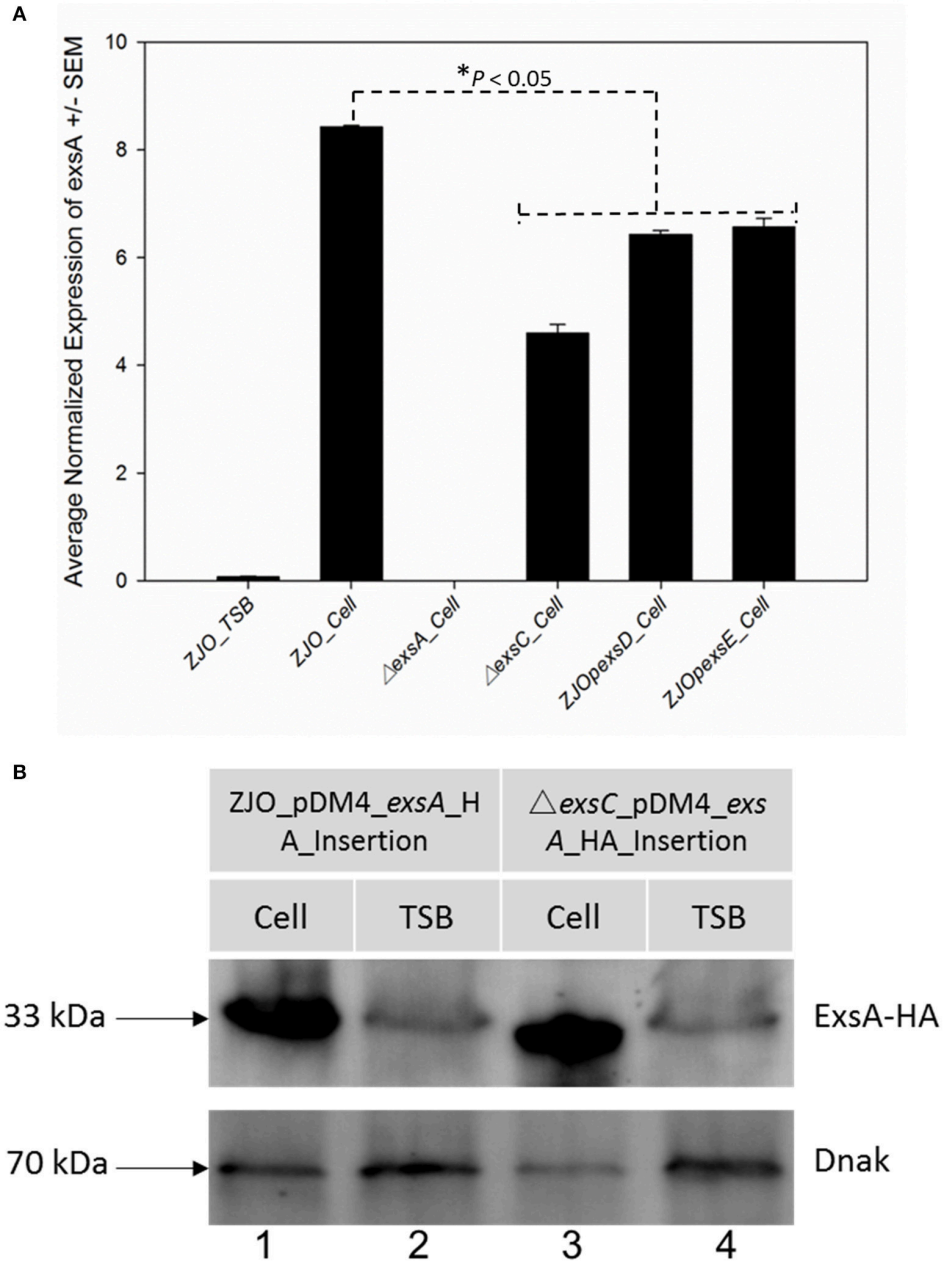
*****exsA*** can be directly activated by alternative signaling pathway. (A)** The transcription of *exsA* was not completely inhibited by deletion of *exsC* or overexpression of *exsD* and *exsE* in wild-type *V. alginolyticus* **(B)** *exsA* has similar expression pattern in Δ*exsC* mutant and wild-type strain when contacted with FHM cells (lane 1, 3) suggesting other factors may also contribute to the regulation of *exsA*. We detected limited expression of *exsA* in non-inducing conditions (lane 2, 4). Endogenous *dnaK* served as a loading control.

Loss of *exsC* or *in trans* expression of *exsD* or *exsE* are associated with reduced transcription of *exsA* (Figure 3A; *P* < 0.05), but the magnitude of *exsA* transcription was still well in excess of what was observed for non-inducing conditions (Figure 3A; *P* < 0.001). This could occur if *exsA* is at least partially autoregulated, but this also raises the possibility that other factors contribute to the regulation of T3SS in *V. alginolyticus*. To determine if protein levels were similarly affected, we quantified the synthesis of ExsA in both wild-type *V. alginolyticus* and the Δ*exsC* mutant strain by adding an *in cis* HA tag the 3′ terminus of ExsA. Western blot analysis indicated ExsA was produced at limited levels under non-inducing conditions (Figure 3B, lanes 2 and 4), but at higher concentrations by both wild-type and Δ*exsC* mutant strains when in contact with host cells (Figure 3B, lanes 1 and 3; *P* > 0.05). Thus, we surmise that a T3SS-independent regulatory pathway exists that can upregulate *exsA* expression independent of the ExsACDE regulon.

### ExsE binds ExsC

**Figure 4:**
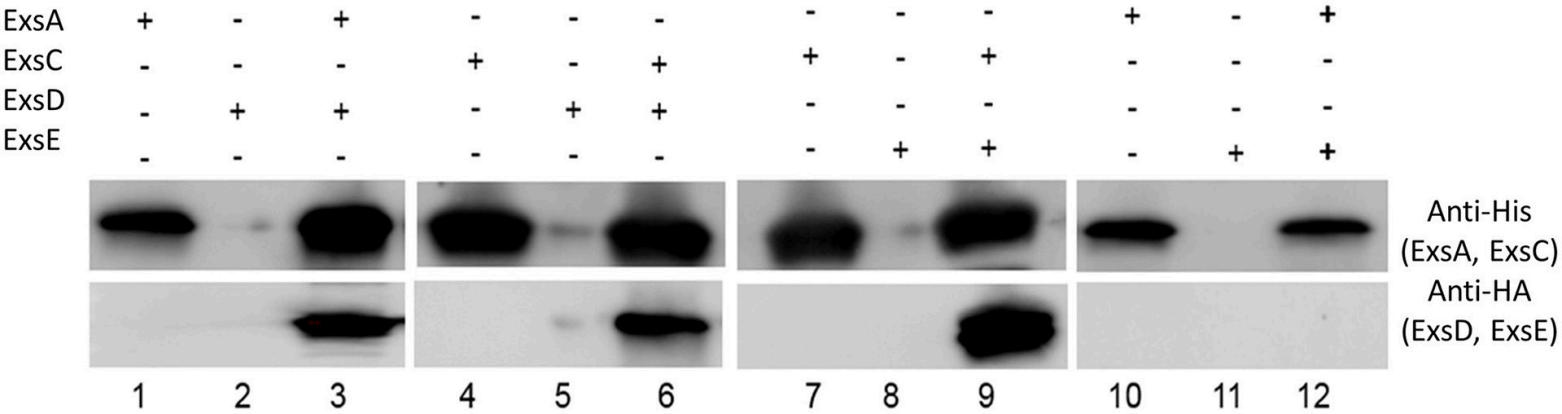
**ExsE interacts with ExsC**. His-tagged ExsA, ExsC and HA-tagged ExsD, ExsE were expressed and incubated overnight at 4°C to examine protein-protein interaction. ExsA binds to ExsD (lane 3), ExsC binds to ExsD (lane 6) and ExsE binds ExsC (lane 9). Non-specific bindings were excluded in this experiment (lane 2, 5, 8, and11) and ExsE does not bind to ExsA (lane 12). + and − indicate the presence and absence of corresponding proteins, respectively.

Co-immunoprecipitation experiments with His-tagged ExsC and HA-tagged ExsE demonstrated that these two proteins likely interact as expected (Figure 4, lane 9). Similar experiments indicated that ExsE does not directly bind to ExsA (Figure 4, lane 12). The expected interactions between ExsA and ExsD (Figure 4, lane 3) and ExsC and ExsD (Figure 4, lane 6) were both confirmed.

### ExsE is secreted under inducing conditions

**Figure 5:**
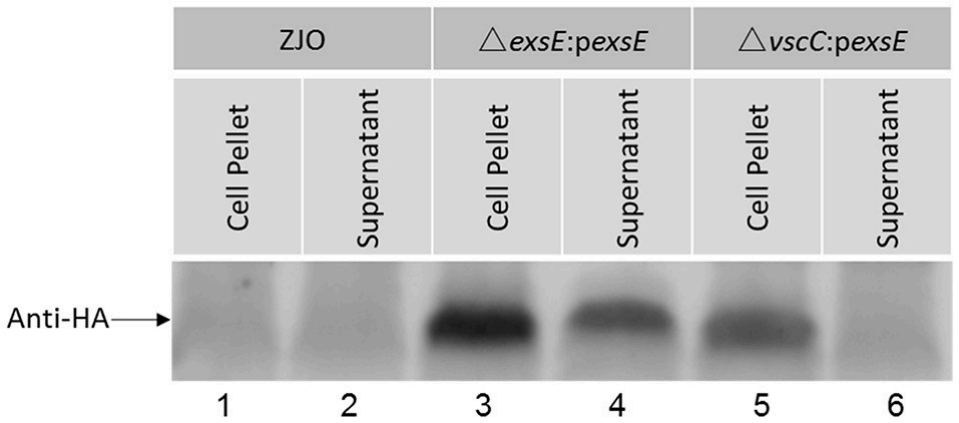
**ExsE is secreted in a T3SS-dependent manner under inducing conditions**. FHM cells were infected with *V. alginolyticus* strains (ZJO, Δ*exsE*:p*exsE* and Δ*vscC*:p*exsE*) and media was collected (4 h), filtered, precipitated and probed. Recombinant ExsE was detected in the supernatant (lane 4) unless a non-function T3SS was present (lane 6).

Western blot analysis indicated that *V. alginolyticus* ExsE was secreted under inducing conditions (Figure 5, lane 4). VscC is a structural protein that is required for a functional T3SS in *V. alginolyticus* (Zhao et al., 2010). When *vscC* was knocked out and *exsE* was expressed *in trans* (Δ*vscC*:p*exsE*), ExsE was not detected in the supernatant (Figure 5, lane 6). Collectively, these results are consistent with T3SS-dependent secretion of ExsE. No proteins bands were detected when the wild-type *V. alginolyticus* was cultured in inducing conditions, confirming the specificity of the antibody that was used for the western blot (Figure 5, lane 1 and 2).

### Physical contact with host cells is not required to trigger expression of T3SS in *V. alginolyticus*

**Figure 6:**
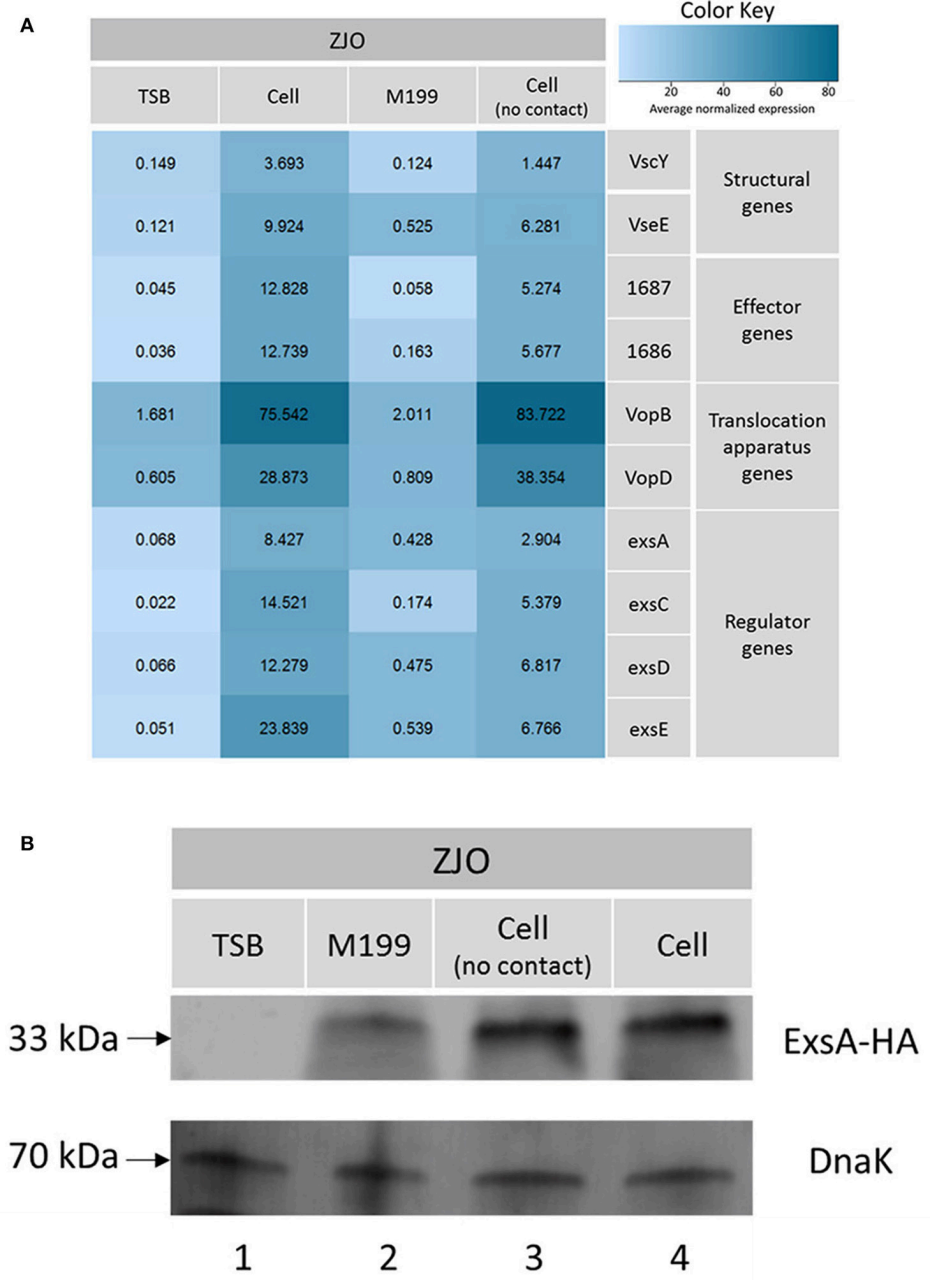
**Physical contact with host cells is not required to induce T3SS in ***V. alginolyticus***. (A)** Transcription of T3SS genes in *V. alginolyticus* was induced when cultured in cell culture media even in the absence of physical contact with eukaryotic cells. TSB and host cell contact served as negative and positive controls in this experiment. Average normalized expression data were added into individual box in the heatmap. **(B)** Without contacting with FHM cells, the expression of master regulator ExsA was induced to a high level (lane 3) which is comparable to host cell model (lane 4). Endogenous *dnaK* served as a loading control.

*Vibrio alginolyticus* was cultured in cell culture media (M199) on a six-well plastic plate (Nunc) with or without polycarbonate membrane inserts. Small molecules can pass through the membrane, but physical contact is blocked when host cells and *V. alginolyticus* are grown on either side of the membrane. Transcription of T3SS genes was upregulated >15-fold when host and bacterial cells were cultured in this manner (Figure 6A) although the effect was not statistically significant relative to M199 alone (*t* = −1.95, 9 df, *P* = 0.08). The lack of statistical significance is probably related to partial upregulation from the M199 media (relative to TSB; *t* = 4.39, 9 df, *P* = 0.002) and dilution of the presumptive soluble factor that signals for upregulation of *exsA*. Qualitatively, it appeared that the abundance of ExsA was similar when ZJO was grow in direct contact with cells or when separated by the membrane (Figure 6B; *P* > 0.05). ExsA was detected when ZJO was cultured in M199 compared to TSB (Figure 6B; *P* < 0.05), which is consistent with the upregulation of T3SS genes in the media alone (Figure 6A).

### ExsE does not contribute to cell adhesion

*Vibrio parahaemolyticus* ExsE is required for adhesion to HeLa cells and a Δ*exsE* mutant strain exhibited a significant reduction in cyto-adherence compared with the wild-type strain (Erwin et al., 2012). We tested if *V. alginolyticus* ExsE contributes to bacterial adhesion to FHM cells following a standard adherence assay (Letourneau et al., 2011). After a 30 min incubation, 24.6% ± 0.03 of Δ*exsE* mutant cells adhered to FHM cells, which was comparable to 23.4% ± 0.06 of wild-type *V. alginolyticus*, indicating ExsE is not required for adhesion to FHM cells.

## Discussion

The type III secretion system (T3SS) gene expression is induced by contact with eukaryotic cells or under specific environmental conditions. A regulatory cascade is usually involved in the transcriptional regulation of T3SS genes with a regulator that activates an AraC-like transcriptional activator. For example, in *P. aeruginosa* T3SS genes are upregulated under low-calcium growth condition (Finck-Barbancon et al., 1997; Vallis et al., 1999) and ExsE serves as a secreted regulator that indirectly contributes to the transcriptional activator ExsA by releasing ExsC to bind to ExsD (Rietsch et al., 2005). This model has been applied to *Vibrio* species and functional orthologues were identified in *V. parahaemolyticus* (Zhou et al., 2008, 2010; Kodama et al., 2010), but the mechanisms involved in the regulation of T3SS genes in *Vibrio* strains remain less clear (Erwin et al., 2012). In this study, we examined the transcription pattern of 10 T3SS genes from *V. alginolyticus* that encode presumptive structural proteins (*n* = 2), effector proteins (*n* = 2), translocation apparatus proteins (*n* = 2) and regulatory proteins (*n* = 4). As expected from *P. aeruginosa* and *V. parahaemolyticus* literature, deletion of *exsE*, or *exsD* resulted in significant increases in constitutive T3SS gene transcription and a similar up-regulation was detected from wild-type *V. alginolyticus* when complemented with either *exsA* or *exsC* (Figure 2 and Figure S2). These data indicated that both ExsA and ExsC are positive regulators while ExsD and ExsE are negative regulators for T3SS genes transcription in *V. alginolyticus*. Interestingly, the deletion of *exsE* enhanced the cytotoxicity of *V. alginolyticus*, which contrasts with the functional orthologue from *V. parahaemolyticus* (Erwin et al., 2012).

ExsE, a secreted repressor of the T3SS regulon that binds to ExsC, is involved in the ExsACDE regulatory cascade in *P. aeruginosa* (Rietsch et al., 2005). We confirmed that *V. alginolyticus* ExsE contributes to regulation of T3SS gene transcription in a manner similar to *P. aeruginosa* (i.e., via a specific interaction with ExsC; Figure 4). Kodama et al. (2010) indicated that *V. parahaemolyticus* ExsE exerted a negative regulatory effect on the production of T3SS1-related proteins. In contrast, Erwin et al. (2012) demonstrated that *V. parahaemolyticus* ExsE has no apparent impact on the synthesis of T3SS1 proteins although it was required for *in vitro* cytotoxicity using a HeLa cell model. To better characterize the role of ExsE in *V. alginolyticus*, we generated an *exsE* deletion mutant and overexpressed this gene in the wild-type *V. alginolyticus*. Consistent with the *P. aeruginosa* ExsACDE model, deletion of *exsE* from *V. alginolyticus* resulted in the greater cytotoxicity toward FHM cells while overexpression of the gene correspondingly inhibited host-cell death (Figure 1). We further examined the T3SS gene transcription and found that overall transcription is curtailed significantly when ExsE is overexpressed (Figure 2). Coupled with the lack of effect on adhesion relative to *V. parahaemolyticus* (Erwin et al., 2012), these differences may be species-specific differences, or they could be due to use of cell lines during infection experiments. The latter scenario is unlikely because in subsequent experiments we found that deletion of *exsE* from *V. alginolyticus* also enhanced the cytotoxicity toward HeLa cells (Figure S3). Erwin et al. (2012) reported reduced cyto-adhesion and swarming motility when *exsE* was deleted in *V. parahaemolyticus* and this is likely due to the loss of flagella biogenesis in the *exsE*-deficient strain. Herein we confirmed that deletion of *exsE* resulted in a reduced swarming phenotype in *V. parahaemolyticus*, but a non-swarming phenotype was observed in both *exsE* deletion mutant and wild-type *V. alginolyticus* (Figure S4). Thus, we surmise the species-specific differences account for these distinct phenotypes.

*Pseudomonas aeruginosa* ExsE is a secreted protein (Rietsch et al., 2005), and translocation of ExsE into Chinese Hamster Ovary cells is required for induction of T3SS gene expression (Urbanowski et al., 2007). We did not detect the translocation of ExsE into FHM cells based on an immunofluorescent assay (data not shown). Limited detection sensitivity of our assay or inherent difference among cell lines may explain this difference. It is also possible that translocation is not required for *V. alginolyticus* to trigger infection because its T3SS can be activated in the absence of direct contact with FHM cells (Figure 6). Indeed, even culture in M199 media is sufficient to induce some upregulation of the T3SS genes (Figure 6).

ExsA is the master transcriptional regulator of T3SS genes in *V. alginolyticus* and it is clearly required for *in vitro* cytotoxicity (Figures S1, S2). Our data demonstrated that ExsD binds to ExsA and ExsC binds to ExsD as expected (Figure 4). Deletion of *exsC* or overexpression of *exsD* or *exsE* did not completely suppress T3SS transcription (Figure 2 and Figure S2), and *exsA* transcription was still significantly higher than under non-inducing conditions (Figure 3A). This could occur if *exsA* is at least partially autoregulated, but also raises the possibility that *exsA* can be regulated by an alternative regulatory pathway that is independent of the T3SS. We further tested this hypothesis by examining the expression of *exsA* both in wild-type *V. alginolyticus* and Δ*exsC* mutant under inducing conditions, and our data revealed that deletion of *exsC* does not reduce the expression of *exsA* when in contact with FHM cells (Figure 3B). The observed phenotype in *V. alginolyticus* is consistent with previous data from *V. parahaemolyticus* (Zhou et al., 2010), but Dasgupta et al. (2004) reported diminished synthesis of ExsA from an Δ*exsC* strain in *P. aeruginosa*. In addition, compared to undetectable expression of *exsA* in LB containing 3% NaCl (non-inducing) from *V. parahaemolyticus* (Zhou et al., 2010), we observed the synthesis of ExsA under non-inducing conditions for *V. alginolyticus* (Figure 3B, lane 2 and 4). The apparent low-level presence of ExsA suggests that extant T3SS structures may be present at all times as has been reported for other pathogens (Cornelis, 2006). These T3SS could serve as “signaling” system for host-cell contact or for detection of soluble signal molecules (Figure 6).

In spite of the distinct differences between *V. alginolyticus, V. parahaemolyticus*, and *P. aeruginosa*, it is clear that the regulatory ExsACDE system is largely conserved for these T3SSs, although differences are apparent for ExsE beyond its contribution to the ExsACDE system. Loss of *exsE* in *V. parahaemolyticus* affects adhesion and cytotoxicity whereas loss of *exsE* in *V. alginolyticus* enhances cytotoxicity. ExsE is secreted by *V. alginolyticus* but its translocation is not required for upregulation of the T3SS as has been reported for *P*. *aeruginosa*. Indeed, co-culture but not physical contact is sufficient to up regulate transcription of *exsA* and thus an external sensing system appears to contribute to the regulation of the *V. alginolyticus* T3SS.

## Author contributions

JL and ZZ conceived the experiments. JL, SL, LO, JA, and ZZ performed the experiments. JL, SL, LO, CR, CH, DC, and ZZ analyzed the results. JL, DC, and ZZ wrote the manuscript. CR, CH, and DC contributed the reagents.

### Conflict of interest statement

The authors declare that the research was conducted in the absence of any commercial or financial relationships that could be construed as a potential conflict of interest.

## Supplementary material

Figure S1 **(A)** ExsA is required for *V. alginolyticus* to induce *in vitro* cytotoxicity. **(B)** ExsC is a positive regulator for T3SS-induced host cell death in *V. alginolyticus*. **(C)** T3SS-induced host cell cytotoxicity is negatively regulated by ExsD in *V. alginolyticus*. Asterisk indicates statistically significant difference (^*^*P* < 0.05; ^**^*P* < 0.01; ^***^*P* < 0.001).

Figure S2 **Transcription pattern of T3SS genes in different ***V. alginolyticus*** strains**. ExsA **(A)** and ExsC **(B)** exhibit positive regulatory effects on transcription of T3SS genes in *V. alginolyticus* and ExsD **(C)** is a negative regulator. Average normalized expression data were added into individual box in the heatmap.

Figure S3 **Deletion of ***exsE*** in ***V. alginolyticus*** enhanced the cytotoxicity toward HeLa cells**. NY4 is the wild-type *V. parahaemolyticus* and LDH assay was assessed after 4 h incubation with HeLa cells. Asterisk indicates statistically significant difference (*P* < 0.05).

Figure S4 **Both ***exsE*** deletion mutant and wild-type ***V. alginolyticus*** were presenting a non-swarming phenotype**. Swarm agar was inoculated with the indicated strains and incubated at 30°C for 8 h. The assay was repeated three times with similar results and a representative photograph is shown here.

## References

[B1] Austin B. (2010). *Vibrios* as causal agents of zoonoses. Vet. Microbiol. 140, 310–317. 10.1016/j.vetmic.2009.03.015 19342185

[B2] Campanelli A. Sanchez-Politta S. Saurat J. H. (2008). Cutaneous ulceration after an octopus bite: infection due to *Vibrio alginolyticus*, an emerging pathogen. Ann. Dermatol. Venereol. 135, 225–227. 10.1016/j.annder.2007.04.010 18374857

[B3] Chen C. Xie J. Hu C. Q. (2009). Phenotypic and genetic differences between opaque and translucent colonies of *Vibrio alginolyticus*. Biofouling 25, 525–531. 10.1080/08927010902964578 19408137

[B4] Chien J. Y. Shih J. T. Hsueh P. R. Yang P. C. Luh K. T. (2002). *Vibrio alginolyticus* as the cause of pleural empyema and bacteremia in an immunocompromised patient. Eur. J. Clin. Microbiol. Infect. Dis. 21, 401–403. 10.1007/s10096-002-0726-0 12072928

[B5] Cornelis G. R. (2006). The type III secretion injectisome. Nat. Rev. Microbiol. 4, 811–825. 10.1038/nrmicro1526 17041629

[B6] Dasgupta N. Lykken G. L. Wolfgang M. C. Yahr T. L. (2004). A novel anti-anti-activator mechanism regulates expression of the *Pseudomonas aeruginosa* type III secretion system. Mol. Microbiol. 53, 297–308. 10.1111/j.1365-2958.2004.04128.x 15225323

[B7] Erwin D. P. Nydam S. D. Call D. R. (2012). *Vibrio parahaemolyticus* ExsE is requisite for initial adhesion and subsequent type III secretion system 1-dependent autophagy in HeLa cells. Microbiology 158, 2303–2314. 10.1099/mic.0.059931-0 22767546 PMC3542814

[B8] Finck-Barbancon V. Goranson J. Zhu L. Sawa T. Wiener-Kronish J. P. Fleiszig S. M.. (1997). ExoU expression by *Pseudomonas aeruginosa* correlates with acute cytotoxicity and epithelial injury. Mol. Microbiol. 25, 547–557. 10.1046/j.1365-2958.1997.4891851.x 9302017

[B9] Gay P. Le Coq D. Steinmetz M. Berkelman T. Kado C. I. (1985). Positive selection procedure for entrapment of insertion sequence elements in gram-negative bacteria. J. Bacteriol. 164, 918–921. 2997137 10.1128/jb.164.2.918-921.1985 PMC214340

[B10] Hauser A. R. (2009). The type III secretion system of *Pseudomonas aeruginosa*: infection by injection. Nat. Rev. Microbiol. 7, 654–665. 10.1038/nrmicro2199 19680249 PMC2766515

[B11] Hueck C. J. (1998). Type III protein secretion systems in bacterial pathogens of animals and plants. Microbiol. Mol. Biol. Rev. 62, 379–433. 9618447 10.1128/mmbr.62.2.379-433.1998 PMC98920

[B12] Kodama T. Yamazaki C. Park K. S. Akeda Y. Iida T. Honda T. (2010). Transcription of *Vibrio parahaemolyticus* T3SS1 genes is regulated by a dual regulation system consisting of the ExsACDE regulatory cascade and H-NS. FEMS Microbiol. Lett. 311, 10–17. 10.1111/j.1574-6968.2010.02066.x 20722736

[B13] Letourneau J. Levesque C. Berthiaume F. Jacques M. Mourez M. (2011). *In vitro* assay of bacterial adhesion onto mammalian epithelial cells. J. Vis. Exp. e2783 10.3791/2783 PMC3197129 21633326

[B14] Livak K. J. Schmittgen T. D. (2001). Analysis of relative gene expression data using real-time quantitative PCR and the 2^−ΔΔCT^ method. Methods 25, 402–408. 10.1006/meth.2001.1262 11846609

[B15] Makino K. Oshima K. Kurokawa K. Yokoyama K. Uda T. Tagomori K.. (2003). Genome sequence of *Vibrio parahaemolyticus*: a pathogenic mechanism distinct from that of *V cholerae*. Lancet 361, 743–749. 10.1016/S0140-6736(03)12659-1 12620739

[B16] Mccaw M. L. Lykken G. L. Singh P. K. Yahr T. L. (2002). ExsD is a negative regulator of the *Pseudomonas aeruginosa* type III secretion regulon. Mol. Microbiol. 46, 1123–1133. 10.1046/j.1365-2958.2002.03228.x 12421316

[B17] Milton D. L. Norqvist A. Wolf-Watz H. (1992). Cloning of a metalloprotease gene involved in the virulence mechanism of *Vibrio anguillarum*. J. Bacteriol. 174, 7235–7244. 10.1128/jb.174.22.7235-7244.1992 1429449 PMC207417

[B18] Milton D. L. O'toole R. Horstedt P. Wolf-Watz H. (1996). Flagellin A is essential for the virulence of *Vibrio anguillarum*. J. Bacteriol. 178, 1310–1319. 10.1128/jb.178.5.1310-1319.1996 8631707 PMC177804

[B19] Morales V. M. Bäckman A. Bagdasarian M. (1991). A series of wide-host-range low-copy-number vectors that allow direct screening for recombinants. Gene 97, 39–47. 10.1016/0378-1119(91)90007-X 1847347

[B20] Niu C. Graves J. D. Mokuolu F. O. Gilbert S. E. Gilbert E. S. (2005). Enhanced swarming of bacteria on agar plates containing the surfactant Tween 80. J. Microbiol. Methods 62, 129–132. 10.1016/j.mimet.2005.01.013 15823402

[B21] Nydam S. D. Shah D. H. Call D. R. (2014). Transcriptome analysis of *Vibrio parahaemolyticus* in type III secretion system 1 inducing conditions. Front. Cell. Infect. Microbiol. 4:1. 10.3389/fcimb.2014.00001 24478989 PMC3895804

[B22] Rietsch A. Vallet-Gely I. Dove S. L. Mekalanos J. J. (2005). ExsE, a secreted regulator of type III secretion genes in *Pseudomonas aeruginosa*. Proc. Natl. Acad. Sci. U.S.A. 102, 8006–8011. 10.1073/pnas.0503005102 15911752 PMC1142391

[B23] Troisfontaines P. Cornelis G. R. (2005). Type III secretion: more systems than you think. Physiology (Bethesda). 20, 326–339. 10.1152/physiol.00011.2005 16174872

[B24] Tseng T. T. Tyler B. M. Setubal J. C. (2009). Protein secretion systems in bacterial-host associations, and their description in the Gene Ontology. BMC Microbiol. 9(Suppl. 1), S2. 10.1186/1471-2180-9-S1-S2 19278550 PMC2654662

[B25] Urbanowski M. L. Brutinel E. D. Yahr T. L. (2007). Translocation of ExsE into Chinese hamster ovary cells is required for transcriptional induction of the *Pseudomonas aeruginosa* type III secretion system. Infect. Immun. 75, 4432–4439. 10.1128/IAI.00664-07 17635873 PMC1951186

[B26] Vallis A. J. Yahr T. L. Barbieri J. T. Frank D. W. (1999). Regulation of ExoS production and secretion by *Pseudomonas aeruginosa* in response to tissue culture conditions. Infect. Immun. 67, 914–920. 9916108 10.1128/iai.67.2.914-920.1999 PMC96404

[B27] Yahr T. L. Wolfgang M. C. (2006). Transcriptional regulation of the Pseudomonas aeruginosa type III secretion system. Mol. Microbiol. 62, 631–640. 10.1111/j.1365-2958.2006.05412.x 16995895

[B28] Zhao Z. Chen C. Hu C. Q. Ren C. H. Zhao J. J. Zhang L. P.. (2010). The type III secretion system of *Vibrio alginolyticus* induces rapid apoptosis, cell rounding and osmotic lysis of fish cells. Microbiology 156, 2864–2872. 10.1099/mic.0.040626-0 20576689

[B29] Zhao Z. Zhang L. Ren C. Zhao J. Chen C. Jiang X.. (2011). Autophagy is induced by the type III secretion system of *Vibrio alginolyticus* in several mammalian cell lines. Arch. Microbiol. 193, 53–61. 10.1007/s00203-010-0646-9 21046072

[B30] Zhou X. Konkel M. E. Call D. R. (2010). Regulation of type III secretion system 1 gene expression in *Vibrio parahaemolyticus* is dependent on interactions between ExsA, ExsC, and ExsD. Virulence 1, 260–272. 10.4161/viru.1.4.12318 21178451 PMC3073295

[B31] Zhou X. Shah D. H. Konkel M. E. Call D. R. (2008). Type III secretion system 1 genes in *Vibrio parahaemolyticus* are positively regulated by ExsA and negatively regulated by ExsD. Mol. Microbiol. 69, 747–764. 10.1111/j.1365-2958.2008.06326.x 18554322 PMC2610376

