# Supplementary figures and images for: ExsE Is a Negative Regulator for T3SS Gene Expression in Vibrio alginolyticus
Source: Front Cell Infect Microbiol. 2016 Dec 6;6:177. doi: 10.3389/fcimb.2016.00177 (PMC5138213; doi:10.3389/fcimb.2016.00177)

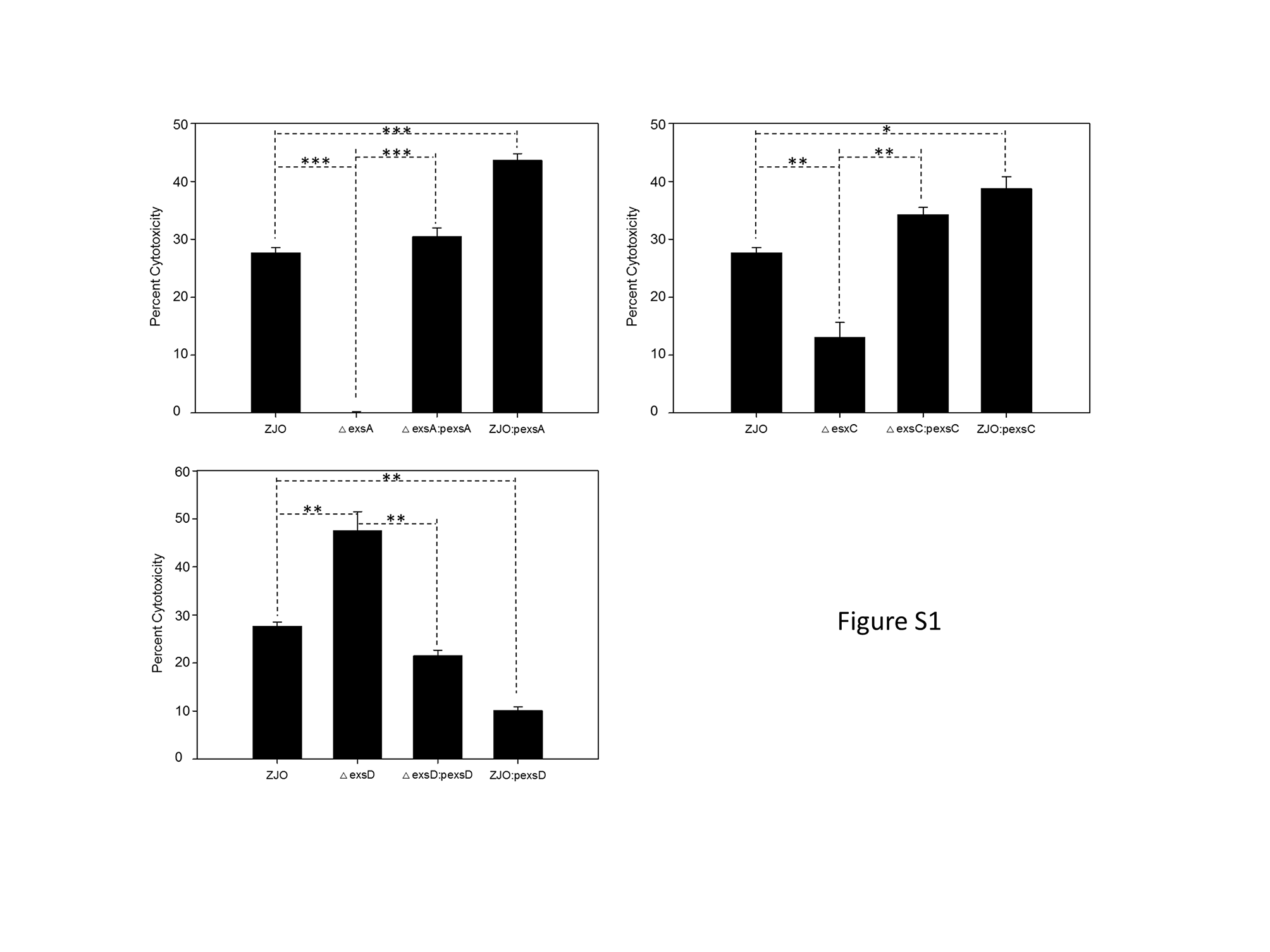

Supplement: Figure S1 — (A) ExsA is required for V. alginolyticus to induce in vitro cytotoxicity. (B) ExsC is a positive regulator for T3SS-induced host cell death in V. alginolyticus. (C) T3SS-induced host cell cytotoxicity is negatively regulated by ExsD in V. alginolyticus. Asterisk indicates statistically significant difference (*P < 0.05; **P < 0.01; ***P < 0.001). [file Image1.tif]

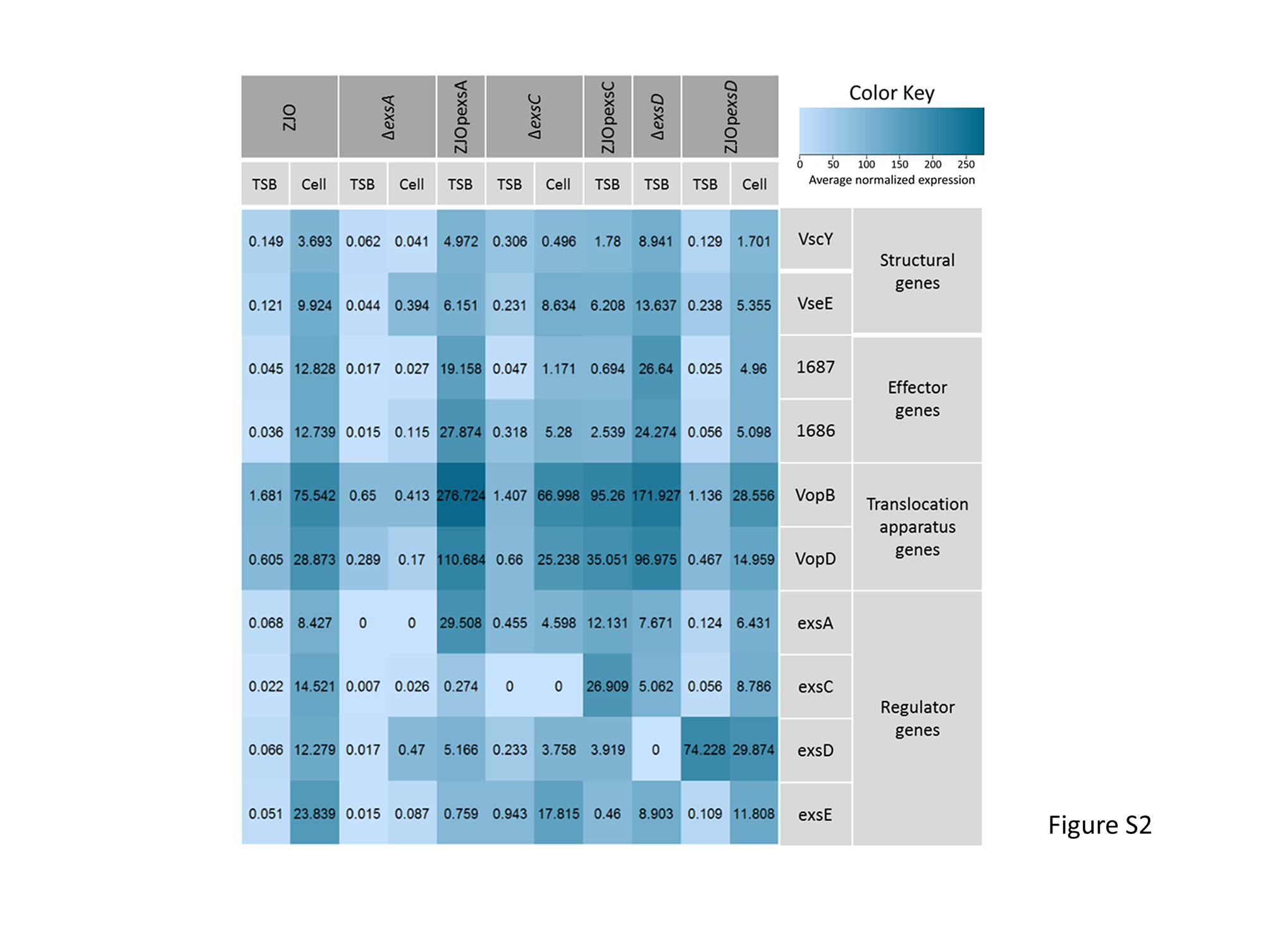

Supplement: Figure S2 — Transcription pattern of T3SS genes in different V. alginolyticus strains. ExsA (A) and ExsC (B) exhibit positive regulatory effects on transcription of T3SS genes in V. alginolyticus and ExsD (C) is a negative regulator. Average normalized expression data were added into individual box in the heatmap. [file Image2.tif]

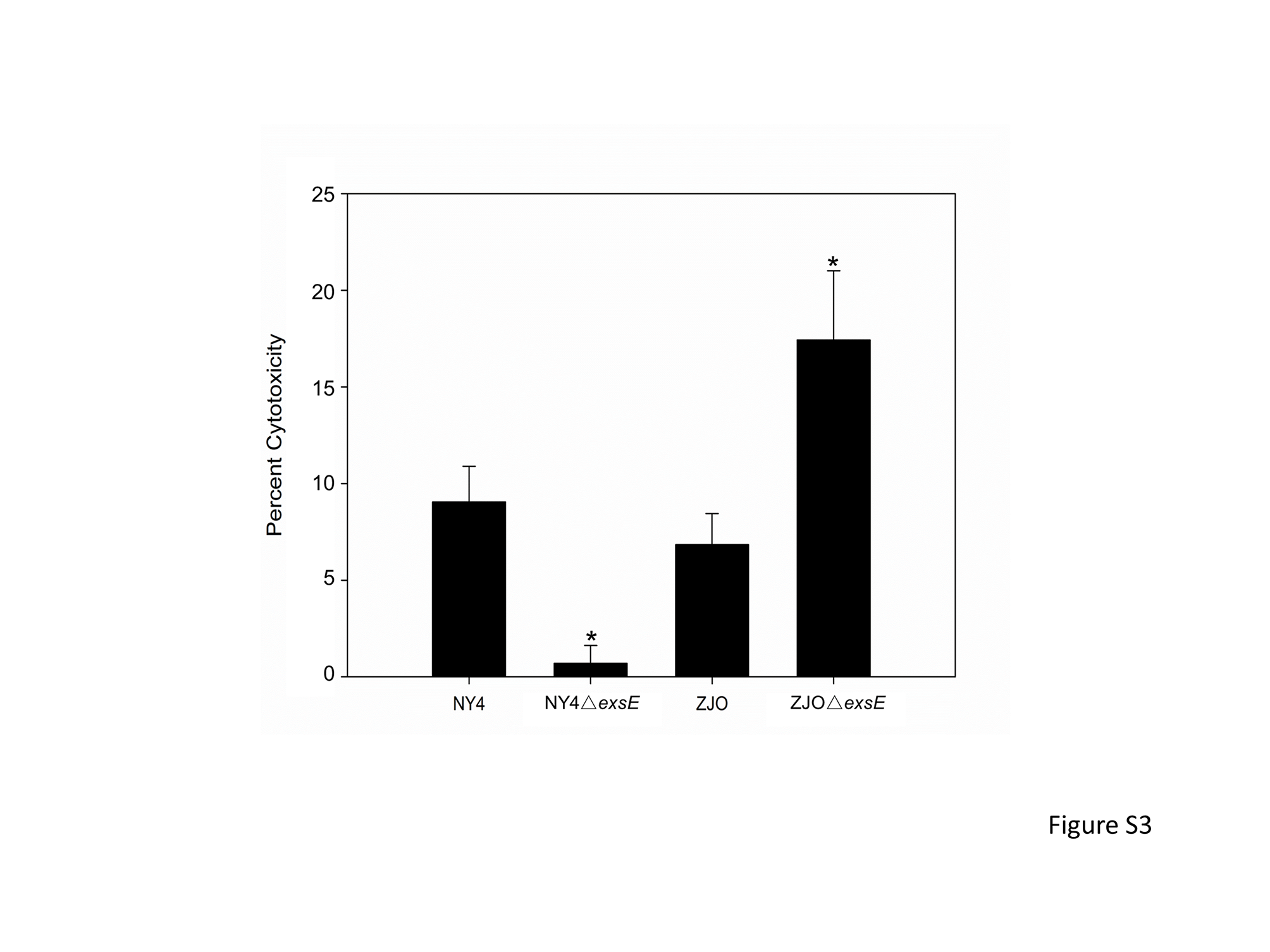

Supplement: Figure S3 — Deletion of exsE in V. alginolyticus enhanced the cytotoxicity toward HeLa cells. NY4 is the wild-type V. parahaemolyticus and LDH assay was assessed after 4 h incubation with HeLa cells. Asterisk indicates statistically significant difference (P < 0.05). [file Image3.tif]

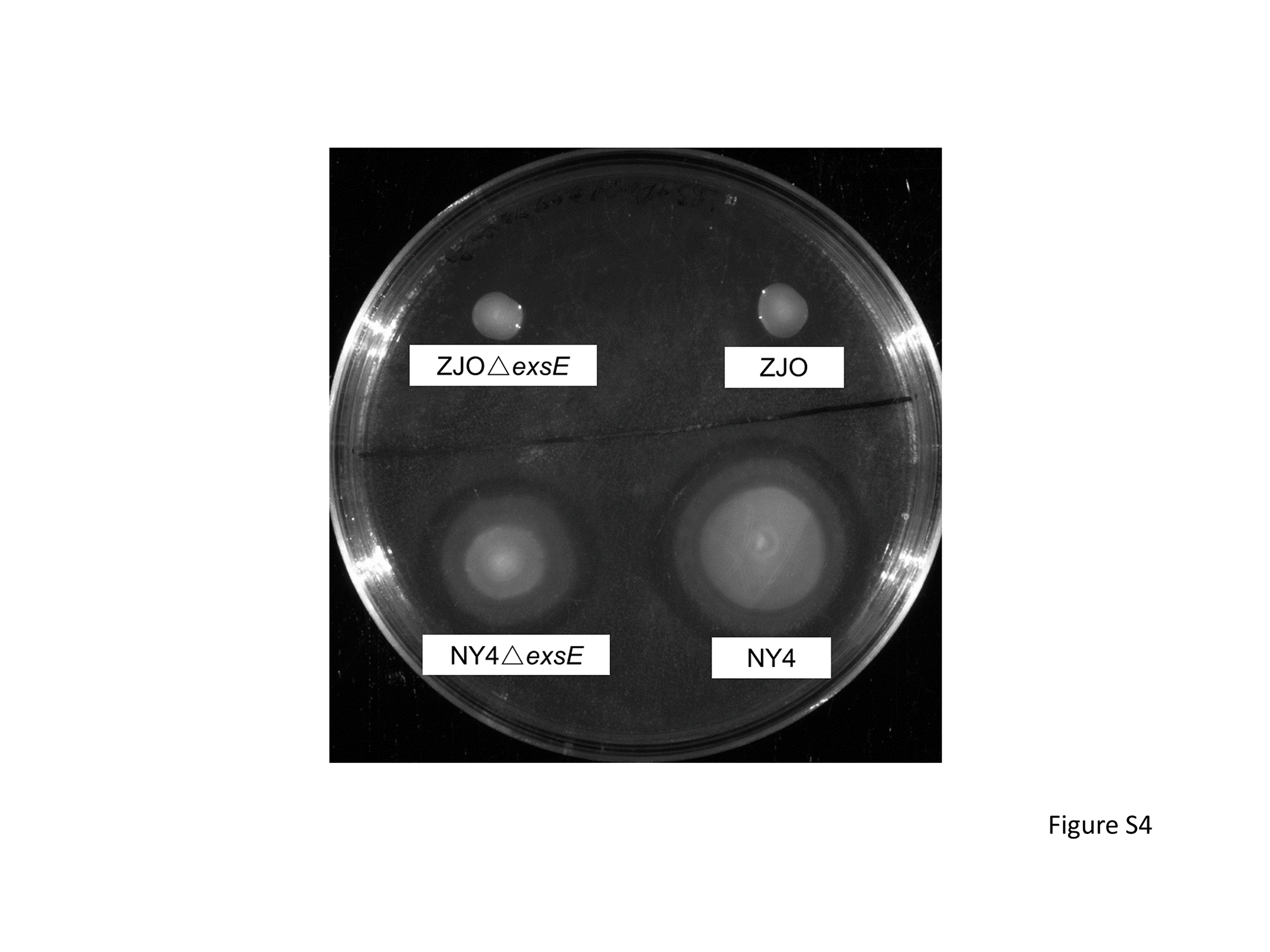

Supplement: Figure S4 — Both exsE deletion mutant and wild-type V. alginolyticus were presenting a non-swarming phenotype. Swarm agar was inoculated with the indicated strains and incubated at 30°C for 8 h. The assay was repeated three times with similar results and a representative photograph is shown here. [file Image4.tif]
